# Repurposing Lenvatinib as A Potential Therapeutic Agent against Thyroid Eye Disease by Suppressing Adipogenesis in Orbital Adipose Tissues

**DOI:** 10.3390/ph15111305

**Published:** 2022-10-22

**Authors:** Lu Cheng, Jing Hu, Ling Zhang, Ning Shen, Hui Chen, Fang Zhang

**Affiliations:** 1 National Clinical Research Center for Eye Diseases, Shanghai General Hospital, Shanghai Jiao Tong University School of Medicine, Shanghai 200080, China; 2 Shanghai Key Laboratory of Fundus Diseases, Shanghai 200080, China; 3 Shanghai Engineering Center for Visual Science and Photomedicine, Shanghai 200080, China; 4 Shanghai Engineering Center for Precise Diagnosis and Treatment of Eye Diseases, Shanghai 200080, China

**Keywords:** thyroid eye disease, Lenvatinib, orbital adipose tissue, vascular endothelial growth factor receptor

## Abstract

Thyroid eye disease (TED) is the most common orbital disease in adults. Targeting expanded orbital adipose tissue (OAT) removed by surgery has therapeutic potential. However, drugs targeting OAT are unavailable because of the lack of deciphering features of OAT. Here, we aimed to investigate the mechanism underlying OAT expansion and identify a drug targeting OAT in TED. We found an increasing number of adipocytes with smaller size in TED-derived OATs as compared with controls, indicating that hyperplasia rather than hypertrophy contributed to OAT enlargement in TED. Typically smaller-sized adipocytes in TED patient-derived OATs were noted to localize surrounding vessels. RNA sequencing revealed enriched vascular endothelial growth factor receptor (VEGFR) genes in adipocytes differentiated from preadipocytes of TED-derived stromal vascular fraction (SVF). Similarly, OATs in patients with TED also expressed a higher level of VEGFR-1 and -2. We induced adipogenesis in TED-derived SVF with or without Lenvatinib, an FDA-approved small-molecule VEGFR inhibitor. Lenvatinib significantly suppressed lipid accumulation in a dose-dependent manner. In conclusion, our study revealed the potential anti-adipogenic effect of Lenvatinib on the OAT of TED-affected patients. In addition to proposing a drug for TED treatment, this study shows the therapeutic potential of anti-adipogenesis drugs targeting the VEGF pathway.

## 1. Introduction

Thyroid eye disease (TED), an autoimmune disorder, is the most common orbital disorder in adults with threats to their sight [1]. TED has a wide spectrum of ocular manifestations, including exophthalmos, conjunctival injection, lid retraction, and diplopia [2]. Most of the signs and symptoms can be explained by an increase in the volume of orbital contents. The expansion of orbital adipose tissues (OATs) [3] and the extraocular muscle [4] can be observed using orbital computed tomography (CT) or magnetic resonance imaging (MRI) [5,6]. The combination leads to proptosis and can, if untreated, result in eyesight complications, including corneal exposure and compressive optic neuropathy [7].

Currently, orbital decompression surgery is an effective approach to treat TED through removing the expanded OAT. Therefore, drugs targeting the expansion of OAT are required for TED patients undergoing surgery as an alternative option. Although the pathophysiology of OAT expansion in TED has not been completely elucidated, studies have demonstrated that orbital fibroblasts (OFs) are the major effector cells causing the disease [8,9]. OFs express both thyrotropin receptor (TSHR) and insulin-like growth factor-1 receptor (IGF-1R) at higher levels than normal fibroblasts [10], resulting in enhanced hyaluronan (HA) production, thereby leading to the enlargement of orbital contents [11]. Novel medications targeting IGF-1R are effective in reducing HA production and alleviating proptosis in TED [12]. However, details of the mechanisms underlying OAT expansion are lacking, and medications targeting OAT expansion are unavailable. 

Generally, the expansion of adipose depots can be driven either by an increase in adipocyte size (hypertrophy) or by the formation of new adipocytes during the process of adipogenesis (hyperplasia) [13], which has been explored in subcutaneous, visceral, and brown adipose tissue depots [14,15]. However, features of adipocytes, including morphology, preadipocyte proliferation, and capacity for adipogenesis, differ among adipose tissue depots. Features in OAT and the mechanisms contributing to the TED phenotype have not yet been explored. It is well known that differentiation from adipocyte precursors (preadipocytes) to adipocytes is governed by a network of transcription factors, including CCAAT/enhancer-binding protein α (*C/EBPα)* and peroxisome proliferator-activated receptor gamma (*PPARγ*). After C/EBPα and PPARγ expression, cells enter the stage of lipid accumulation and lipid droplet formation with the expression of adipocyte fatty acid binding protein 4 (*FABP4*), adiponectin (*ADIPOQ),* and Perilipin 1 (*PLIN1*) [16]. In vitro co-culturing of TED OFs with PPARγ ligands led to adipogenesis, indicating that adipogenesis is associated with OATs expansion [17,18]. This was also evidenced by increased expression levels of adipocyte-specific genes leptin, ADIPOQ, fatty acid synthase, FABP4, and PPARγ mRNA in TED-affected adipose tissue compared with normal orbital tissue [19]. However, the underlying molecular mechanism and the role of hyperplasia in TED have not been clarified. 

Adipogenesis and angiogenesis are temporally and spatially coupled processes that reciprocally interact via paracrine signaling systems throughout adult life. The adipose tissue stroma contains blood vessels and other cell types. It is well documented that preadipocytes among stromal cells can be induced to differentiate into adipocytes, and inhibition of angiogenesis reduces adipose tissue mass [20,21]. These findings strongly suggest that angiogenesis plays a key role in adipogenesis. Vascular endothelial-derived growth factor (VEGF) plays an important role in vasculogenesis and angiogenesis. VEGF receptor (VEGFR) -1 and -2 are the main signaling receptors [22]. It has been reported that anti-VEGF treatment inhibits not only angiogenesis, but also adipogenesis in obese animals [23]. However, little is known about the relationship between adipogenesis and angiogenesis in TED and the role of the VEGF pathway in adipogenesis in TED.

Therefore, in the current study, OAT samples from patients with TED and control participants were examined, and the size and number of adipocytes were analyzed to explore the role of hypertrophy and hyperplasia in OAT expansion in TED. The features of the distribution of adipocytes in perivascular regions were also revealed. RNA sequencing (RNA-Seq) analysis of the OATs was performed to identify the underlying molecular links between adipogenesis and angiogenesis in OAT expansion in TED. Based on the above links, we found a novel medication candidate targeting adipogenesis.

## 2. Results and Discussion

### 2.1. Contribution of Hyperplasia to Oat Expansion in TED

The size of adipose tissue can be increased in two main ways: hypertrophy (existing adipocytes increase in size) or hyperplasia (formation of new adipocytes through differentiation of resident precursors known as preadipocytes) [13,14,15]. The number of adipocytes in a given adipose depot was believed to be determined early in life and remain stable throughout adulthood [24]. However, an increasing number of studies have demonstrated that under some special conditions such as prolonged excess intake of calories new adipocytes can emerge from the differentiation of preadipocytes and contribute to adipose tissue expansion [25]. Similarly, in vitro studies have demonstrated that OFs derived from patients with TED can be induced to undergo adipogenesis as preadipocytes [17,18,26] and that TED-affected OAT expressed higher levels of adipocyte-specific genes [19] indicating that adipogenesis is associated with TED-affected OAT expansion. However, whether hypertrophy also occurs during OAT expansion has not been explored.

**Figure 1:**
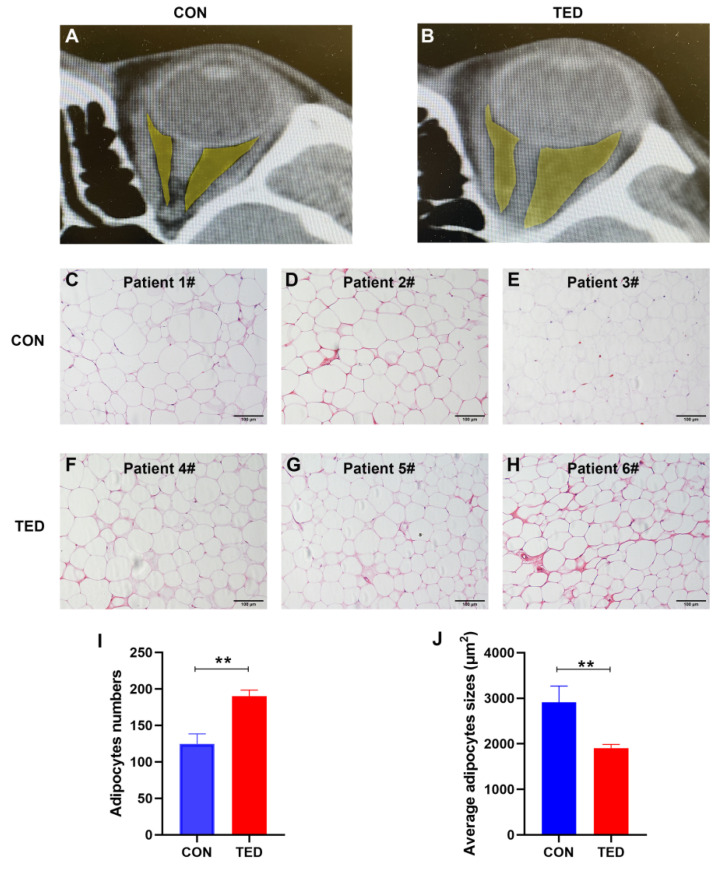
Hyperplasia rather than hypertrophy contributes to the enlargement of orbital adipose tissues (OATs) in thyroid eye disease (TED). Representative images of ocular computed tomography of patients with TED (**A**) shows an increased volume of OATs (indicated by yellow color) compared with the controls (**B**). Representative photographs (20×) of OATs derived from controls (**C**–**E**) and patients with TED (**F**–**H**) are shown. (**I**) In the quantification of adipocyte numbers, four fields were randomly selected from each section at a constant magnification (20×), and their average was calculated. There were more adipocytes in sections from TED-derived OATs (190.0 ± 7.6 cells/field) as compared with the controls (124.7 ± 11.3 cells/section). (**J**) Quantification of adipocyte sizes showed that TED-derived OATs had smaller adipocytes (1904 ± 72.34 μm^2^/cell, n = 1000 cells) compared with controls (2908 ± 293.9 μm^2^/cell, n = 1000 cells). ** *p* < 0.01 as compared to the controls.

To study the role of hypertrophy and hyperplasia in OAT expansion under TED conditions, we obtained OAT histological sections that included adequate numbers of adipocytes from five patients with TED (three female and two male) and three control participants undergoing surgical decompression. The mean age of the patients was 36.6 (range: 28–68) years. Among the three controls, one was treated for congenital exophthalmos (female/31 years), and two were treated for orbital cavernous hemangioma (female/36 years and female/52 years). As shown in Figure 1, we found that the volume of OATs in the five patients with TED increased obviously as compared to the controls (Figure 1A,B), indicating that expansion of OATs occurred. Meanwhile, TED-affected OATs (Figure 1F–H) consisted of smaller adipocytes compared with the controls (Figure 1C–E). We further analyzed the sizes and numbers of adipocytes, and found that TED OATs had a significantly higher number of adipocytes (control 124.7 ± 11.3 and TED 190.0 ± 7.6 cells/field; *p* < 0.01) (Figure 1I) with smaller sizes compared with those of the controls (control 2908 ± 293.9 and TED 1904 ± 72.34 μm^2^/cell; n = 1000 cells in each group, *p* < 0.01; Figure 1J). The appearance of small new adipocytes could be reflective of the high capacity for adipocyte hyperplasia. Therefore, these observations indicate that TED-induced OAT expansion is mainly due to hyperplasia rather than hypertrophy.

### 2.2. Perivascular Distribution of Smaller-Sized Adipocytes in OAT in TED

Activated adipocytes produce multiple angiogenic factors that either singularly or collectively stimulate angiogenesis during fat mass expansion [27]. Rodent studies have demonstrated a relationship between angiogenesis and adipogenesis; small adipocytes are always provided with a supply of blood vessels. Coupled angiogenesis is essential for adipogenesis in the development of obesity [23]. Thus, anti-angiogenic agents provide a novel therapeutic option for the prevention and treatment of human obesity and related disorders [28]. However, the relationship between adipogenesis and angiogenesis in TED has not been explored.

**Figure 2:**
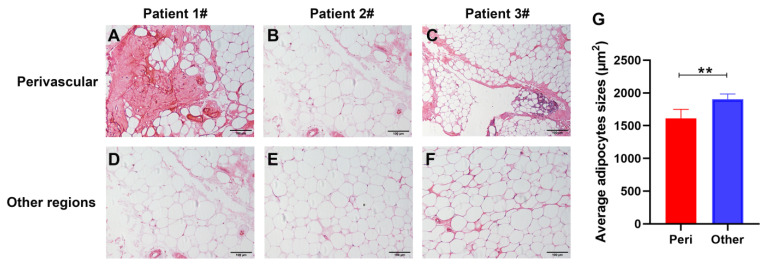
Perivascular adipocytes were smaller than adipocytes in other locations in thyroid eye disease (TED)-derived orbital adipose tissues (OATs). Representative photographs (20×) of perivascular sections (**A**–**C**) showed smaller adipocytes compared with sections far away from vessels (**D**–**F**) in TED-derived OATs. (**G**) Quantification of average adipocyte sizes showed that perivascular adipocytes were significantly smaller than adipocytes in other regions (n = 1000 cells in each group, ** *p* < 0.01 as compared to the controls).

In our study, hematoxylin and eosin (H&E)-stained sections from TED-affected OATs were used to investigate the spatial relationship between blood vessels and adipocytes. The results showed that the adipose tissue surrounding the fine networks of capillaries consisted of smaller adipocytes (Figure 2A–C) compared with the adipose tissue located far away from the blood vessels (Figure 2D–F). The average size of the adipocytes was significantly smaller in perivascular regions than in other regions (1610.46 ± 123.9 and 1904.4 ± 72.3 μm^2^/cell, respectively; n = 1000 cells in each group, *p* < 0.01; Figure 2G). Notably, these findings demonstrated that angiogenesis was related to adipogenesis in orbital adipose deposits of TED, which is consistent with the findings of earlier studies on obesity.

### 2.3. Role of the Vascular Endothelial Growth Factor Receptor Pathway in Adipogenesis 

**Figure 3:**
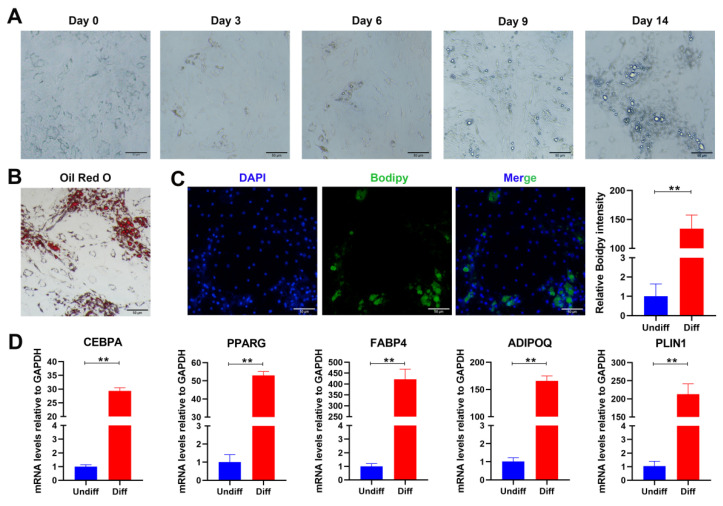
In vitro adipocyte differentiation from preadipocytes of thyroid eye disease (TED)-derived stromal vascular fraction (SVF). (**A**) Cells derived from the TED-affected stromal vascular fraction (SVF) treated with adipogenic medium at days 0, 3, 6, 9, and 14. (**B**) Intracellular lipids of cells treated with adipogenic medium were stained with Oil-Red-O on day 14. (**C**) Lipid droplets in differentiated adipocytes were identified with BODIPY409/503 stain on day 14, and the bodipy intensity of differentiated cells was significantly higher than that of undifferentiated cells. (**D**) Expression of adipocyte-specific genes in differentiated adipocytes and undifferentiated preadipocytes on day 7. (n = 3 in each group, ** *p* < 0.01 as compared to the controls).

Adipogenesis is a complex process by which preadipocytes transform into adipocytes. To analyze the potential interaction between adipogenesis and angiogenesis at the cellular level, which may mediate the orbital fat hyperplasia seen in TED, we developed an in vitro adipocyte differentiation model. In this model, cells derived from the TED-affected stromal vascular fraction (SVF) were treated with rosiglitazone, dexamethasone, and 3-isobutyl-1-methylxanthine (IBMX) to induce adipocyte differentiation. On day 9 after initiation, a subpopulation of preadipocytes became rounded and was surrounded with lipid droplets; on day 14, there was a major population of rounded adipocytes surrounded by numerous lipid droplets (Figure 3A). Lipid accumulation was observed on day 14 through Oil-Red-O staining (Figure 3B). BODIPY409/503 staining of the fixed cultures was used to assess the degree of adipocyte differentiation (Figure 3C). Generally, adipogenesis is initiated by the induction of *C/EBPα* and *PPARγ,* which in turn induce the expression of genes involved in terminal adipocyte differentiation, including *FABP4*, *ADIPOQ,* and *PLIN1* [26,27]. In our study, the mRNA levels of these adipocyte-specific markers were significantly increased on day 7 in differentiated adipocytes compared with undifferentiated preadipocytes (Figure 3D), demonstrating the developmental progression from orbital preadipocytes through adipocyte stem cells to mature adipocytes.

**Figure 4:**
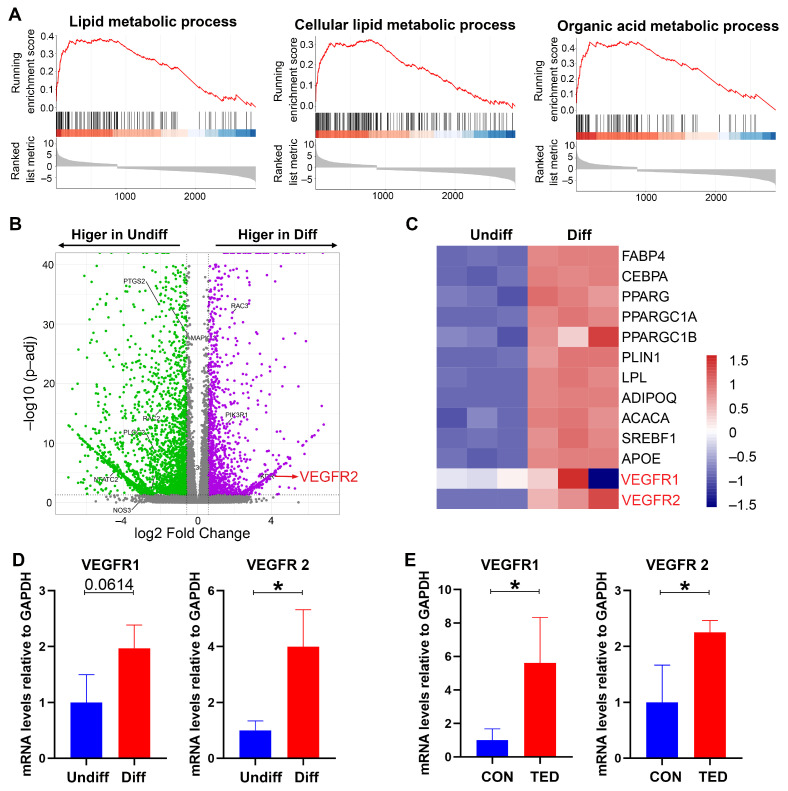
RNA-Seq analysis comparing TED-derived differentiated adipocytes and undifferentiated preadipocytes. (**A**) GSEA analysis showing pathways that were enriched in the differentiated adipocytes group. (**B**) Volcano plot of genes that were higher in differentiated adipocytes or undifferentiated preadipocytes. (**C**) Heatmap demonstrating gene expression differences between the differentiated and undifferentiated group. (**D**) Gene expression level in differentiated and undifferentiated cells examined by qRT-PCR on day 7. (**E**) Gene expression level in orbital adipose tissues (OATs) of controls and patients with TED examined by qT-PCR. * *p* < 0.05 as compared to the controls.

To identify genes involved in adipogenesis of TED, we used RNA-Seq to generate genetic profiles of differentiated adipocytes and undifferentiated preadipocytes from TED-derived SVF as described above. Based on histological changes and adipocyte-specific gene expression in cells treated with adipogenic media, we analyzed the gene expression of differentiated adipocytes and undifferentiated preadipocytes on day 7. GO-analysis of differentially expressed genes was performed using Gene Set Enrichment (GSEA). It revealed a marked upregulation of the cellular lipid metabolic, lipid metabolic, and organic acid metabolic processes in differentiated adipocytes (Figure 4A). A volcano plot revealed that the gene expression patterns of the differentiated and undifferentiated TED-derived cells were segregated (Figure 4B). We observed 1064 genes that were enriched in differentiated adipocytes, including *KDR*, *PIK3R1*, and *RAC3,* as well as 912 genes that were enriched in undifferentiated preadipocytes, including *MAPK, PTGS2, RAC2, PLCG2,* and *NFATC2* (Figure 4B). Notably, *KDR*, the gene encoding VEGFR-2, was enriched in differentiated cells (Figure 4B). Heat map analysis of the expressed genes demonstrated significant differences between differentiated cultures compared with undifferentiated cultures (adjusted *p* < 0.05; Figure 4C). Moreover, heat map analysis of individual genes within the VEGF pathway demonstrated the level of enrichment of the genes *KDR* and *FLT1* (the gene encoding VEGFR-1) on differentiated adipocytes (Figure 4C). The significantly increased expression of *KDR* (*p* < 0.05) and an increasing tendency of *FLT1* gene expression (*p* = 0.0614) were also verified via qRT-PCR (Figure 4D). 

To further verify the role of the VEGF pathway in TED-induced adipogenesis in vivo, the mRNA expression levels of VEGFR-1 and VEGFR-2 in OATs from patients with TED and controls were tested. The results revealed increased expression levels of both VEGFR-1 and VEGFR-2 in TED-affected OAT compared with those in normal orbital tissue (n = 3 in each group, *p* < 0.01; Figure 4E). As the key participants in the VEGF signaling pathway, increased mRNA expression of VEGFR-1 and VEGFR-2, which were found both in adipocytes differentiated from preadipocytes of TED-derived SVF and in the TED-affected OATs, indicated that the VEGF pathway may participate in adipogenesis and may be the molecular links between adipogenesis and angiogenesis in the condition of TED.

During postnatal development in obese animals, adipose stromal cells and recruited inflammatory cells secrete high levels of VEGF, significantly contributing to adipose angiogenesis, showing a correlation between adipogenesis and obesity [28,29]. Another rodent study revealed that VEGF and VEGFB play counteractive roles in adipose differentiation, in which VEGFB inactivation causes expansion of white adipose tissue, whitening of brown adipose tissue, and an increase in fat accumulation; in contrast, VEGF repression induces expansion of brown adipose tissue and brown adipocyte development in white adipose tissue [30]. In addition, an in vitro study of 3T3-L1 preadipocytes showed that inhibiting VEGFB suppressed adipogenesis [31]. These studies show that the role of the VEGF pathway may differ among different types of adipose tissue depots. Our study has demonstrated that VEGFR-1 and VEGFR-2 may play consistent promoting roles in the orbital adipogenesis of TED and could be a potential therapeutic target for TED by inhibiting adipogenesis.

### 2.4. Effects of Lenvatinib, A VEGFR Inhibitor, on Adipogenesis 

VEGFR mediates initial interactions between blood vessels and adipocyte precursors [32,33]. Notably, although anti-VEGF treatment does not affect the average size of large adipocytes, it markedly inhibits the formation of small differentiating adipocytes as well as the formation of blood vessel sprouts and adipogenic/angiogenic cell clusters [23].

**Figure 5:**
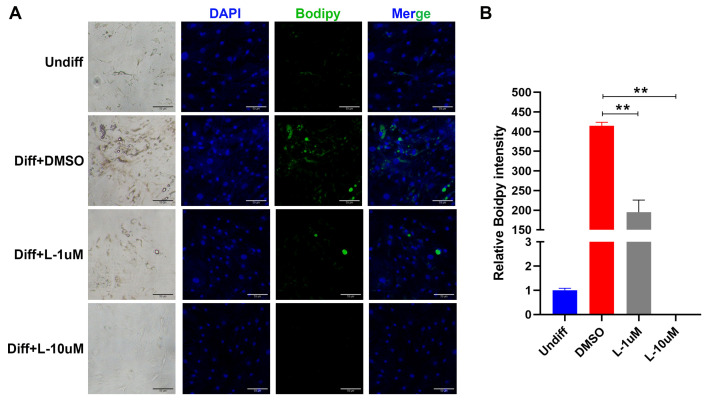
Anti-adipogenesis effect of Lenvatinib. (**A**) Cells derived from the TED-affected stromal vascular fraction (SVF) were induced to adipogenic differentiation. These cells were treated with 1 or 10 μM of Lenvatinib, or DMSO as a vehicle control in induction media. After 14 days of adipogenic differentiation, intracellular neutral lipids were evaluated with BODIPY409/503 staining. (**B**) Lipid accumulation in differentiated adipocytes was inhibited by Lenvatinib in a dose-dependent manner (n = 3 in each group, ** *p* < 0.01).

The RNA-Seq and qRT-PCR results of cells and tissues described above indicated that VEGFR may play a promoting role in the orbital adipogenesis of TED. To verify this point, we induced adipogenesis of TED patient-derived SVF as described above with or without Lenvatinib, an FDA-approved small-molecule inhibitor of VEGFR [34]. Lenvatinib was added to the medium on day 0 after initiation, and its effects on adipocyte differentiation were observed via lipid accumulation measured by BODIPY409/503 staining on day 14. The results showed that Lenvatinib significantly suppressed lipid accumulation in a dose-dependent manner. An inhibition of 53% lipid accumulation at a concentration of 1 µM and 100% at 10 µM was observed (Figure 5). 

These results clearly show that in TED-affected OATs, anti-VEGF treatment inhibits the differentiation of adipocytes, which provides direct evidence that the VEGF pathway plays a key role in orbital adipogenesis in TED. Furthermore, the potential anti-adipogenic effect of Lenvatinib indicates that it can be repurposed for use as a therapeutic agent in the treatment of TED.

## 3. Materials and Methods

### 3.1. Sample Collection and Patients

All participants involved in this study signed written informed consent forms as approved by the Medical Ethical Committee of Shanghai General Hospital (approval reference number: 2021KY008) and adhered to the Declaration of Helsinki regarding research involving human subjects. Orbital adipose tissue (OAT, predominantly white fat) was obtained from patients with TED undergoing surgical decompression and from control participants with normal fat level who were treated for other non-inflammatory conditions. Samples were snap-frozen in liquid nitrogen and stored at –80 °C until use for cryostat sectioning. In all instances, the tissue cultures were kept moist on physiological saline-soaked gauze and maintained on ice until the disaggregation process began (<24 h). 

### 3.2. Quantification of Adipocyte Size and Number

We obtained OAT histological sections that included an adequate number of adipocytes from five patients with TED and three control participants. Sections were stained with H&E and captured as digital images via microscopy. Images of the regions of interest were segmented from each section at constant magnification. Four sections were randomly obtained from each sample and four fields were randomly selected from each section, and the average adipocyte number and size were analyzed using AdipoCount (version 1.0, http://www.csbio.sjtu.edu.cn/bioinf/AdipoCount/ (accessed on 15 August 2022) and ImageJ (National Institutes of Health, Bethesda, MA, USA).

### 3.3. In-Vitro Differentiation

OAT explants were obtained from patients with TED who underwent surgical decompression. The patients were inactive, with a CAS of < 4 at the time of surgery. Explants were washed with penicillin–streptomycin–amphotericin B Solution (Beyotime Biotechnology) and minced into pieces smaller than 1 mm. Tissue fragments were digested with 175 U/mL Collagenase, Type 1 (Diamond, Cat# A004194) at 37 °C for 2 h. Digestion was stopped with 10% fetal bovine serum in DMEM/F12, and red blood cells were lysed with Red Blood Cell Lysing Buffer (BD Biosciences). The supernatant was discarded, and the obtained SVF in the tube bottom was resuspended in DMEM/F12 supplemented with 10% FBS and 1% penicillin–streptomycin (PS). Cells were incubated at 37 °C in a humidified environment with 5% CO_2_. The medium was changed every three days until the cells reached confluency. Strains were stored in liquid N_2_ until needed and used between passages 7 and 15.

Preadipocytes of SVF were induced to undergo adipogenesis. Briefly, fibroblasts between passages seven and fifteen were seeded on plastic tissue culture plates and allowed to proliferate to reach near confluence in DMEM:F-12 (1:1, #11320033; ThermoFisher Scientific, Waltham, MA, USA) containing 10% FBS and PS. The cells were then treated with adipogenic medium consisting of DMEM:F-12 (1:1, #11320033; ThermoFisher Scientific) supplemented with 10% FBS, 5 μg/mL insulin (#12585014; ThermoFisher Scientific), 1 μmol/L rosiglitazone (#R2408; Sigma, St. Louis, MO, USA), and for the first 6 days, 1 μmol/L dexamethasone (#D4902; Sigma, St. Louis, MO, USA) and 0.15 mmol/L IBMX (#13630S; Cell Signalling Technology, Danvers, MA, USA). The medium was changed every three days. The cultures were maintained in the adipogenic medium for 14 days. Control cultures were treated with DMEM:F-12 (1:1) supplemented with 10% FBS, PS, and the vehicle. On day 14, for neutral lipid staining, the media were removed and cells were washed with Phosphate Buffered Saline (PBS) two times. Samples were then fixed with 4% paraformaldehyde (PFA) for 30 min at room temperature and washed three times with PBS. The intracellular lipid was stained using Oil-Red-O following the manufacturer’s protocol (BioVision, Cat# K580). Cells were then observed under a microscope (Nikon, Tokyo, Japan) after being washed four or five times in PBS. 

### 3.4. Immunofluorescence and Imaging

Lipid droplets in differentiated adipocytes were identified with BODIPY409/503 stain (D3922, Invitrogen, Carlsbad, CA, USA), and the nuclei were identified by 4′,6-diamidino-2-phenylindole (DAPI, Beyotime Biotechnology, Cat# 1002) staining following the manufacturer’s protocol. Adipocytes were fixed with 4% PFA and washed three times with PBS. Samples were then incubated with 5 μg/mL BODIPY and 1 0μg/mL DAPI in PBS. After incubation in the dark for 30 min, the samples were washed four or five times with PBS, and images were captured using a Nikon Eclipse Ti-U fluorescence microscope (Tokyo, Japan). The ImageJ software (National Institutes of Health, Bethesda, MA, USA) was used to quantify the Bodipy signal of each image.

### 3.5. RNA-Seq

To compare the gene expression profile in OFs by RNA-Seq analysis, we isolated total RNA of undifferentiated OFs and differentiated OFs using TRIzol Reagent (#15596018; ThermoFisher). To analyze proliferation-related and adipogenesis genes, the construction of an RNA-seq library was completed by Xuran Biotechnology Co. Ltd. (Shanghai, China). The raw sequencing data was processed by the Illumina base-calling pipeline, and the quality of the data was evaluated by FastQC. The clean reads were aligned to the GRCh38 human genome using STAR. The expression levels of the transcripts were calculated by FPKM. Unbiased gene expression analysis profiled the molecular characteristics between undifferentiated and differentiated OFs. The thresholds for determining differentially expressed genes were a *p*-value < 0.05 and an absolute fold change ≥2.

### 3.6. Quantitative Real-Time Polymerase Chain Reaction (qRT-PCR)

Total RNA was extracted from OFs or OF-CL using the RNA Pure Micro Kit following the manufacturer’s protocol (Zymo, Cat# R1005). RNA sample quality and concentration were assessed using a NanoDrop 2000c spectrophotometer (ThermoFisher Scientific). RT Master Mix (Vazyme, Cat# HY-K0510) was used to reverse-transcribe RNA to cDNA, and the ViiA^TM^ 7 Real-Time PCR System (Applied Biosystems) was used to amplify cDNA with a program consisting of 40 cycles of amplification. The 2-ΔΔCt method was used to calculate the gene expression level of each gene. Glyceraldehyde-3-phosphate dehydrogenase (GAPDH) was used to normalize the data. The primers used for real time PCR were: FLT1 primer (forward primer: GAGAGCATCACTCAGCGCAT; reverse primer: GAAGCTTGTAGGTGGCAACA); KDR primer (forward primer: CGGTCAACAAAGTCGGGAGA; reverse primer: CAGTGCACCACAAAGACACG); CEBPa primer (forward primer: AACACGAAGCACGATCAGTCC; reverse primer: CTCATTTTGGCAAGTATCCGA); PPARg2 primer (forward primer: TTCTTTTAACGGATTGATCTTTTGC; reverse primer: TGTCAACCATGGTCATTTCTTGT); FABP4 primer (forward primer: ACTGGGCCAGGAATTTGACG; reverse primer: CTCGTGGAAGTGACGCCTT); PLIN1 primer (forward primer: GCGGAATTTGCTGCCAACACTC; reverse primer: AGACTTCTGGGCTTGCTGGTGT); ADIPOQ primer (forward primer: CAGGCCGTGATGGCAGAGATG; reverse primer: GGTTTCACCGATGTCTCCCTTAG), and GAPDH primer (forward primer: CATGAGAAGTATGACAACAGCCT; reverse primer: AGTCCTTCCACGATACCAAAGT).

### 3.7. Treatment and Quantification of Adipocytes

The validation of the Lenvatinib inhibition on adipogenesis was carried out at a final concentration of 1 or 10 μM of Lenvatinib or an equivalent volume of DMSO as a vehicle control in induction media. After 14 days of adipogenic differentiation, intracellular neutral lipids were evaluated with BODIPY 409/503 staining and quantitative fluorescence image analysis as mentioned above or Oil Red O staining. Briefly, 4% paraformaldehyde-fixed cells were washed three times in phosphate buffered saline (PBS). To stain the lipid droplets, cells were washed with 60% isopropanol and stained with freshly prepared 0.3% (*w/v*) Oil Red O (Sigma) at room temperature for 15 min. The cells were rinsed with 60% isopropanol and then rinsed with PBS. Images were captured using a light microscope (Nikon Eclipse Ti-U, Tokyo, Japan).

### 3.8. Statistical Analysis

For experiments performed in triplicate, the error bars in the figures represent the standard error of the mean (SEM). One-way ANOVA with Tukey’s post hoc test was performed using the GraphPad Prism 5 (GraphPad Software, La Jolla, CA, USA) software for statistical analysis of the effects of the drugs on proliferation or adipogenesis. Statistical significance was considered when *p* < 0.05.

## 4. Conclusions

This study shows that TED-induced OAT expansion is mainly due to hyperplasia rather than hypertrophy of adipocytes, and differentiation of adipocytes is tightly coupled with angiogenesis, which relies on the VEGF/VEGFR signaling pathway. Notably, our study is the first to report the relationship between adipogenesis and angiogenesis in human OATs in TED and to reveal the role of VEGFR promoting orbital adipogenesis of TED. The present study also demonstrated the suppressive effect of Lenvatinib on adipogenic differentiation, revealing the potential of Lenvatinib in exerting its anti-adipogenic activity on the OAT in TED. Our findings allow us to confidently suggest repurposing Lenvatinib as a potential therapeutic agent in TED; however, further studies are needed to clarify how inhibition of the VEGF pathway leads to the reduction of OAT and the underlying molecular mechanism. Therapeutic treatments centered on anti-adipogenesis targeting the VEGF pathway are likely to emerge for TED in the future.

## Data Availability

Data is contained within the article.
